# NeuroCOVID-19: a critical review

**DOI:** 10.1590/0004-282X-ANP-2022-S136

**Published:** 2022-08-12

**Authors:** Bruno Fukelmann Guedes

**Affiliations:** 1 Universidade de São Paulo, Faculdade de Medicina, Hospital das Clínicas, Departamento de Neurologia, São Paulo, SP, Brazil.

**Keywords:** COVID-19, Cognitive Dysfunction, Patient Participation, Social Media, COVID-19, Disfunção Cognitiva, Participação do Paciente, Mídias Sociais

## Abstract

**Background:**

The COVID-19 pandemic has challenged neurologists since its early days. Neurology consultation services were then overloaded by emergency department and intensive-care patients with acute neurological syndromes. These complications are better explained today, but the growing number of patients with reported longstanding neurological symptoms constitute an emerging, complex, and still poorly understood phenomenon.

**Objective:**

This review summarizes data on relevant neurological manifestations of acute SARS-CoV-2 infection and lasting post-infectious disease, also known as Long COVID. The complex history of Long COVID is examined to illustrate the upsides and challenges imposed by the active participation of patient communities in the production of medical knowledge.

**Methods:**

Narrative review.

**Results:**

Infection with the severe acute respiratory syndrome coronavirus 2 is associated with encephalopathy/delirium, cerebrovascular disease, headache, and peripheral nervous system involvement. Long COVID is a living concept jointly defined by patient communities, physicians and scientists, including neurologists.

**Conclusion:**

Co-production of Long COVID knowledge between scientists and patients has initiated an era of patient-led research and evidence-based activism that acts as a two-edged sword - putting patient’s suffering in the spotlight, but with a tradeoff in methodological consistency.

## RESUMO

**Antecedentes:**

A pandemia de COVID-19 trouxe desafios aos neurologistas desde seus primeiros dias. Os serviços de interconsulta neurológica foram inicialmente sobrecarregados pelo volume de atendimentos a síndromes neurológicas agudas em departamentos de emergência e unidades de terapia intensiva. Essas complicações são bem conhecidas hoje, mas o verdadeiro desafio está no número crescente de pacientes com sintomas atribuídos a “Long COVID”/COVID Longa, um problema novo, complexo e ainda pouco compreendido.

**Objetivo:**

Esta revisão resume literatura sobre manifestações neurológicas da infecção aguda por SARS-CoV-2 e COVID longa. A complexa história de COVID longa é examinada para ilustrar os ganhos e as dificuldades impostos pela participação ativa das comunidades de pacientes na produção de conhecimento médico.

**Métodos:**

Revisão narrativa.

**Resultados:**

A infecção pelo SARS-CoV-2 está associada a encefalopatia/delirium, doença cerebrovascular, cefaléia e neuropatias periféricas. COVID Longa é um conceito ainda plástico, definido em conjunto por comunidades de pacientes, médicos e cientistas, incluindo neurologistas.

**Conclusão:**

A co-produção do conhecimento sobre COVID longa, unindo cientistas, médicos (incluindo neurologistas), e pacientes, iniciou uma nova era de pesquisa conduzida por pacientes e “ativismo baseado em evidências”. Este atua como uma faca de dois gumes, alinhando os rumos da pesquisa às demandas e sofrimentos dos pacientes, ao custo de problemas metodológicos.

## INTRODUCTION

Since the emergence of coronavirus disease 2019 (COVID-19) in Wuhan, China, the illness caused by severe acute respiratory syndrome coronavirus 2 (SARS-CoV-2) soon spread across the globe, creating an unprecedented pandemic. As of late March, 2022, over 6 million people have died from the disease (https://covid19.who.int/). Brazil, the United States and India are among the countries with the largest death tolls. Many survivors have developed chronic systemic and neurological symptoms, which are building up to an extreme global and personal burden of disease^1^. This review summarizes data on relevant neurological manifestations of acute SARS-CoV-2 infection and lasting post-infectious disease, also known as Long COVID, along with its intricate history.

## NEUROLOGICAL COMPLICATIONS ASSOCIATED WITH ACUTE SARS-COV-2 INFECTION

Although COVID-19 was initially described as a primarily respiratory disease, the respiratory symptoms were more-than-often accompanied by systemic compromise, including neurological complications^2^ ^,^ ^3^, and the pandemic defied neurology services worldwide^3^. 

About one third of patients with COVID-19 experienced at least one neurological symptom^4^.

### Encephalopathy

Encephalopathy is a prominent neurological finding in acute COVID-19. An array of agitation, consciousness abnormalities, delirium or disexecutive syndrome occur in 7.5 - 28% of hospitalized patients^2^ ^,^ ^5^, and in as many as 14.8 - 69% of ICU-admitted patients^2^ ^,^ ^6^. It is associated with higher mortality rates^5^. Encephalopathy has been very demanding for clinicians and intensive care physicians, which is demonstrated by the increased workload of neurology consultation services throughout the course of the pandemic^3^. Encephalopathy and delirium are common in critically ill patients, and COVID-19 shares many clinical characteristics of other acute viral and respiratory conditions such as H1N1 pneumonia and acute respiratory distress syndrome^7^. However, COVID-19-associated encephalopathy is novel, unexpectedly severe, and associated with prolonged conscious abnormalities following sedation withdrawal, which has prompted efforts into identifying potential disease-specific mechanisms. Several studies report MRI, EEG and CSF findings in COVID-19 encephalopathy^8^ ^,^ ^9^. Although some case reports have suggested SARS-CoV-2 may cause infectious and encephalitis^10^ ^,^ ^11^, the evidence for classical encephalitis mechanisms is limited. Most studies on laboratory markers of COVID-19-encephalopathy reported that CSF only rarely shows pleocytosis (less than 10% of CSF samples) or CNS-restricted oligoclonal bands (0-2%)^8^ ^,^ ^12^. Likewise, SARS-CoV-2 RNA is rarely detected (0/76 samples in Jarius et al.^12^; 1/21 in Tuma et al.^8^). Magnetic resonance images usually show non-specific changes in the cerebral white-matter, although unsuspected strokes were also common^8^ ^,^ ^13^. 

### Peripheral neuropathy

Disorders of the peripheral nervous system (PNS) are common in hospitalized patients with acute COVID-19, although their study is limited by the difficulties in performing electrodiagnostic studies and neurological examination (clouded by encephalopathy and sedation), two fundamental diagnostic tools, during the acute stage of the disease. Moreover, many studies evaluating neurological symptoms during acute COVID reported the prevalence of unspecific findings such as “motor weakness” or “sensory abnormalities” without clear nosological diagnoses^8^ ^,^ ^14^, and a subset of the patients with these symptoms may have developed neuropathies. 

A few distinct phenotypes have been described: Guillain-Barré syndrome (GBS); axonal polyneuropathy; and focal neuropathies (single or multiple mononeuropathies, plexopathies). The association between GBS and COVID-19 has been extensively debated^15^ ^,^ ^16^. Many case reports of GBS were published in the early stages of the pandemic, suggesting a potential causal association between SARS-CoV-2 infection and GBS^15^, as frequently seen with other infections. Caress et al. reviewed 37 published case reports from 2020. Patients developed GBS a mean of 11 days after the onset of COVID-19 symptoms. Electrodiagnostic studies showed a demyelinating pattern in half of cases, and serum anti ganglioside antibodies were absent in 15/17 patients tested^15^. A later epidemiological study from the United Kingdom found no association between the SARS-CoV-2 outbreak and the incidence of GBS^16^. GBS is also allegedly related to COVID-19 vaccines, but the true incidence of COVID-19-vaccine-associated GBS may be much lower than expected, and lower than GBS from influenza vaccines^17^. COVID-19 can also cause diffuse weakness and sensory abnormalities suggesting a myopathy/polyneuropathy overlap^18^. 

Focal neuropathies involving nearly every major peripheral nerve from the lower and upper limbs have been described as COVID-19 complications^19^ ^-^ ^23^. Focal neuropathies are usually compressive, and are strongly associated with the severity of acute disease (Bruno F. Guedes, personal communication). Foot drop from uni- or bilateral peroneal nerve injuries are very common and may persist up to 11 months post-COVID in 5% of hospitalized patients (Bruno F. Guedes, personal communication). Internists and neurologists are well-acquainted with peroneal nerve injuries in critically-ill patients. However, an array of known but rare upper limb neuropathies (especially ulnar nerve at elbow level and brachial plexus injuries) caused by mechanical ventilation re-emerged^24^ ^,^ ^25^. Some reports suggest focal neuropathies in COVID can be immune-mediated, based on clinical rationale and a predominance of axonal neuropathies on electrodiagnostic studies^19^ ^,^ ^26^. However, given the rarity of immune-mediated neuropathies and the strong association of focal neuropathies with mechanical ventilation and prone positioning, those could as well be variations of compressive neuropathies. 

### Stroke

Acute cerebrovascular diseases are not uncommon in patients with COVID-19, with an incidence of 1.4%. Ischemic strokes outnumber brain hemorrhage in a 4:1 ratio. The incidence of stroke is much higher when only hospitalized patients are considered, and the disease is strongly associated with COVID-severity^2^ ^,^ ^6^. The real incidence of cerebrovascular disease could be much higher, as several factors may contribute to its underestimation, including limited access to neuroimaging imposed by safety protocols (decontamination of tomogrophers, restrictions to patient circulation), resource shortage in crowded hospitals, and limitations to neurological examination in comatose and sedated patients^27^. This limitation may account for the high proportion of unsuspected strokes presenting as encephalopathy^3^. 

COVID-19 stroke patients were older, and more likely to have hypertension, diabetes mellitus and severe infection. Compared to stroke patients without COVID-19, they were younger, were more likely affected by large vessel occlusion, and had more severe strokes, as evidenced by higher NIHSS scores and much higher in-hospital mortality rates^28^. Interestingly, COVID-19 stroke cases are more likely to be classified as cryptogenic or to involve multiple vascular territories^28^ ^,^ ^29^, suggesting a potential contribution of prothrombotic state^30^. Optimal management of COVID-19 cryptogenic stroke is uncertain. 

### Neuro COVID-19 case reports

Although usually considered low-quality evidence^31^, case reports are particularly relevant at the dawn of pandemics, and should not be neglected^32^. The first case report on the influenza of 2009 in Mexico^33^ was an important whistle-blower that drew attention towards a yet-to-become devastating pandemic^34^. Some case reports are among the most cited publications from the Middle East Respiratory Syndrome period^35^.

There is an ever-growing list of diseases allegedly associated with COVID-19, which includes esoteric case reports describing SARS-CoV-2 - varicella-zoster meningitis coinfection^36^, COVID-19-associated vestibulopathy^37^, nasopharyngeal swab-associated carotid-artery dissection with retinal ischemia^38^, normal pressure hydrocephalus^39^, cerebral vasculitis^40^, and many other rare neurological syndromes. This list even includes diseases with well-known pathophysiological mechanisms, such as neurodegenerative^41^, metabolic^42^, autoimmune^43^, and infectious^44^ diseases. Many of these reports make the case for a theoretical COVID-19-specific disease mechanism in the discussion sections, with mechanicist rationales based on laboratory studies or theoretical reasoning, and with very limited support from clinical studies. The average quality of COVID-19 case reports is very low - a systematic review of 196 case reports showed the adherence to CARE guidelines^45^ was poor, with a mean composite score for completeness of reporting^46^ of 54.%, much lower than the proposed threshold of 75%^47^.

Low-quality case reports are not unique to the COVID-19 pandemic^47^, but the rate of incomplete reports is too high. This is potentially dangerous because the overall completeness of recording does not correlate with article citation or social media exposure, which may lead to harmful biases^47^. In the author’s opinion, the limits of case reports have been dangerously bent, and most COVID-19 case reports are of very limited value. As we transition from the fear of novelty towards a better understanding of the disease, case reports should be repositioned as low-level evidence, and rarely guide clinical decisions.

## THE HISTORY OF LONG COVID

### Evidence-based activism

The designation “long COVID” is now an established concept worldwide. However, the disease’s clinical features and even its naming are still being debated. The emergence of Long COVID as a clinical entity has initiated a new era in the production of medical knowledge. Widespread internet use and social media-empowered patients, and medical knowledge production is now increasingly influenced by patient-generated data, which relies largely on subjective evidence. Rabeharisoa and colleagues defined the concept of “evidence-based activism” to describe “modes of activism that focus on knowledge production”^48^. With evidence-based activism, patient communities seek acknowledgement for their subjective knowledge and collective personal experiences, with the aim of identifying areas of “undone science”. This should then guide government agents and academia towards overlooked but clinically relevant issues. Ultimately, this leads to the “co-production” of knowledge - the role of the scientists as guardians of scientific knowledge is replaced by an ill-defined partnership in which the actions of academia, politics and public opinion are intermingled^49^. 

The COVID pandemic was noticeable for high mortality and a complex acute disease that brought entire healthcare systems to their knees^50^. In the early stages of the pandemic, political, clinical and scientific efforts were all targeted towards reductions in mortality and acute complications. Papers on neurological complications of COVID focused on hospitalized patients with severe acute disease, and emphasized encephalopathy, stroke, neuromuscular disorders and other complex manifestations such as encephalitis^2^ ^,^ ^3^ ^,^ ^8^. Clinicians soon noticed surviving patients were challenged by lasting symptoms and disability, but most scientific efforts towards understanding these difficulties focused on cohort studies of surviving severe COVID patients^51^. Undiagnosed or “mild” COVID patients were largely unstudied. A large mass of unattended patients with chronic symptoms, allegedly COVID-related, gathered around social media and started pushing for recognition. 

### The controversial meaning of long COVID

Long COVID is probably the first illness defined by patients on Twitter^52^ ^,^ ^53^. The term “long COVID” was first proposed by Elisa Perego, an archeologist from Lombardy (a hard- and early-hit region of Italy), in a Twitter publication in late May 2020^54^. The hashtag #LongCovid was used as a contraction of the wording “long-term Covid illness”. Perego became an activist for recognition and validation of a large and growing group of patients suffering from persistent and debilitating symptoms following COVID-19^55^. In June, 2020, #LongCovid’s reach grew as rising patient collectives around the world translated the term to French (#apresJ20), Spanish (#covid persistente), Japanese (#長期微熱組), Finnish (#koronaoire), and several other languages (as seen in patient-organized websites: https://www.apresj20.fr; https://apuakoronaan.fi). 

“Long Covid” first hit the organized press in late June, following the report of how an intensive care physician, Dr Jake Suett, joined a patient-created support group on Facebook^56^. A month later, the British Medical Journal (BMJ) issued a blog post signed by a collective of affected doctors entitled “Patients’ experiences of “longcovid” are missing from the NHS narrative”^57^, which introduced the term to traditional scientific venues. Until mid-2020, there was a clear disconnect between official health communications and Long COVID sufferers online. Online discussions about lasting symptoms of COVID started as early as January-February 2020, and the #LongCOVID and #Long Hauler hashtags gained traction in May-June 2020. A structured analysis of social media content from COVID long haulers showed that “Before the start of June 2020, Long Haulers were (...) invisible: typical tweets reported Long Haulers either being misdiagnosed/’gaslighted’ by their GPs (...) received very little support, information and treatment”^58^. The gap between patients and the medical establishment started to close during the second semester of 2020, when scientific journals and governments started to broadcast Long-COVID content^58^.

The choice of the word “Long” is a novelty. Although “Long COVID” is compliant with WHO naming standards (short, neutral), temporality is hazy. Long COVID proponents felt the usual (and WHO-endorsed) “post-” or “chronic” COVID was inadequate for several reasons. First, it could prevent patients without positive acute-phase COVID tests from being diagnosed. Secondly, “post-” and “chronic” suggest the disease clearly occurs following acute COVID, whereas patient experiences suggested a continuation between acute (or suspected acute) disease and persisting symptoms^52^. 

### Physicians as patients

As part of the COVID crisis frontline, doctors were highly exposed, and the number of physicians suffering from lingering symptoms grew. Their physical and mental health was profoundly impacted by the pandemic^59^. The lack of solid research and science-curated knowledge on the subject prompted the mobilization of affected doctors into filling the knowledge gap with anecdotal reports and personal experience. This is very important because the involvement of doctors as patient-advocates grants technical validity to the claims of advocacy groups. Many patient-physician manifestos were published in important journals^60^ ^,^ ^61^, including that signed by 39 doctors entitled “persistent symptoms of Covid-19 to be treated with a scientific methodology without bias”^60^. This manifesto is a good illustration of this new (and bias-prone) approach to the role doctors can play in the creation of medical knowledge, by combining reports of personal experience with their inherent technical authority, as seen in the following excerpt: “We write as a group of doctors affected by persisting symptoms [...]. We aim to share our insights from both a personal experience of the illness and our perspective as physicians”^60^. 

### Limitations of current definitions of Long COVID

Several different definitions of Long COVID were proposed, with vague clinical criteria and variations mostly in the timing of symptoms (range, 1 month - 24 weeks post-COVID)^1^. The World Health Organization assigned an emergency use of the International Classification of Diseases, Tenth Revision (ICD-10), to classify cases of ‘Post-COVID conditions, unspecified’^62^. Based on a Delphi consensus, the WHO stated ‘post COVID-19 occurs in individuals with a history of probable or confirmed SARS-CoV-2 infection, usually 3 months from the onset of COVID-19 with symptoms that last for at least 2 months and cannot be explained by an alternative diagnosis. (…) Common symptoms include fatigue, shortness of breath, cognitive dysfunction but also others (...) may be of new onset following initial recovery (...) or persist from the initial illnessinstagram post from computer, may fluctuate or relapse (…)’^63^. 

This definition has several limitations, including difficult-to-define time-windows for asymptomatic patients, lack of clinical data to define clinical thresholds, and very low specificity. This is potentially linked to the way co-production took place in the development of Long-COVID definitions.

Patient-driven evidence-based activism was crucial in drawing attention towards the issue of Long COVID, which was inadvertently neglected by the medical establishment in the early stages of the pandemic (see section *The history of “Long COVID* above). However, the fear of ‘falling through the cracks’, and the need for validation of many suffering patients dictated the deliberate creation of inclusive criteria, so no patients would be left out, a legitimate priority^52^ ^,^ ^53^ ^,^ ^55^. Although the current definitions are encompassing, allowing all suffering patients to receive care, the lack of clear diagnostic hallmarks could make clinical studies difficult to perform. The current consensus is slowly shifting away from some of the prerogatives proposed by patient advocacy groups. The explicit exclusion of acute COVID-19 infection is now considered a must in the diagnosis of Long COVID^64^, which contrasts with advocates’ proposal of Long COVID as an acute-chronic symptoms continuum^52^ ^,^ ^53^. 

## “NEURO-LONG-COVID”

Neurological manifestations are high in the list of potential lasting complications of COVID. Almost one third of patients are eventually diagnosed with a neurological or psychiatric illness up to six months post-acute COVID^65^. Although several neurological symptoms and syndromes related to the peripheral (PNS) or central (CNS) nervous system have been described, many such syndromes are likely to be related to dysfunctions in other organ systems. Nonspecific symptoms such as ‘brain fog’, fatigue and sleep disturbances may be associated with CNS, PNS, respiratory, endocrine, cardiovascular, psychiatric or autoimmune diseases^66^. There is great variability in prevalence estimates of neurological symptoms across studies (including cohort studies and patient-led research), which highlights the challenges brought by the lack of standardization, selection and reporting biases^1^. The reported rates for neurological symptoms is high across studies. In a cohort study of 1733 patients with confirmed COVID-19, three quarters reported a history of at least one of the following symptoms: fatigue or muscle weakness (63%), disorders of smell (11%) or taste (7%), headache (2%), mialgias (2%). Anxiety/depression was reported by 23% of patients^67^. Another study of 2433 patients showed similar results^68^, with fatigue (30% of patients) as a leading symptom. A large online survey by the Patient-Led Research Collaborative found far higher prevalences of neurological symptoms including cognitive dysfunction (85%), headache(77%), mood disorders (88%), and memory impairment (77%), which may reflect the intrinsic selection bias of online surveys targeted at patient support groups^68^. A meta-analysis including data from nearly 50,000 patients revealed 80% of patients reported at least one persistent symptom, with headache (44%), fatigue (58%) and attention disorder (27%) as leading complaints^69^.

Headache and cognitive dysfunction are reviewed in greater detail below.

### Headache

Headache is among the most common neurological symptoms in outpatient clinics and emergency departments. It is a very frequent symptom during the acute phase of COVID-19, with 11-34% of hospitalized patients reporting headaches^70^. COVID-19-associated headache lacks specific diagnostic criteria, but to some extent fits the International Classification of Headache disorders-3 (ICHD-3) criteria for *headache attributed to systemic viral infection:*

“(…) caused by and occurring in association with other symptoms and/or clinical signs of a systemic viral infection. Evidence of causation demonstrated by at least two of the following: (1) headache has developed in temporal relation to onset of the systemic viral infection, (2) headache has significantly worsened in parallel with worsening of the systemic viral infection, (3) headache has significantly improved or resolved in parallel with improvement in or resolution of the systemic viral infection, and (4) headache has either or both of the following characteristics: (a) diffuse pain and (b) moderate or severe intensity”^71^. However, this classification does not account for all the particularities of COVID-19 associated headache, and recent research has allowed the definition of COVID-19-headache as a unique headache syndrome. The headache associated with acute COVID-19 is more common in women and younger patients, responds poorly to analgesics, is associated with anosmia/ageusia, and has a mean duration of two weeks^72^. A quarter of patients experience a migraine-like headache, but the most common presentation is tension-type-headache^73^. The pathogenesis of headache in acute COVID is still debatable. Some authors have suggested headache could be caused by proinflammatory cytokines, as indirectly implied from the association of headache with fever, but studies reporting the association between fever and headache showed conflicting results, with two studies showing independence between headache and fever^72^ and another reporting higher odds of having headache in febrile COVID-19 patients^74^. Headache may also be explained by direct invasion of the CNS, as other coronaviruses are known to penetrate the CNS. Moreover, SARS-CoV-2 can infect neurons *in vitro*, and the association between headache and other neurological symptoms such as anosmia strengthens this hypothesis^7^ ^,^ ^72^. Proposed pathways for neurotropism include trans synaptic transmission through the olfactory bulbs and a hematological route, through the blood-brain barrier^7^. 

Headache is also a leading complaint of patients with Long COVID. Although the typical headache duration is two weeks, 16% of patients had persistent headache at 9 months post-infection^75^. Proposed mechanisms of disease in persistent headache are similar to those suggested for acute infection. The clinical and nosological relationship between Long COVID headache and the recently described New Persistent Daily Headache cannot be overlooked, and points towards an inflammatory post-infectious mechanism^76^. Although several immune, infectious and metabolic mechanisms have been proposed early in the pandemic, some key environmental factors were described much later. It is important to acknowledge the unique aspects of the epidemic context in which patients developed headaches. Environmental factors include use of personal protective equipment^77^, worsening of underlying headache disorders following vaccination^78^, and the psychological impact of the pandemic on the daily life of headache patients^79^. Evidence on treatment options remains anecdotal. Although small case series suggest benefits for unorthodox treatments such as indomethacin and sphenopalatine ganglion block^80^, a symptom-oriented approach is usually useful, with patients benefiting from treatment targeted at their headache phenotype.

### Cognitive deficits

Cognitive dysfunction and cognitive complaints are very common in both cohorts of hospitalized patients^81^ and online surveys^68^. A systematic review showed attention disorder, anxiety, brain fog and memory issues were each observed in roughly 25% of patients^82^. Interestingly, subjective symptoms were more common long- (≥6months) than short- (3-6 months post-COVID) term, with a pooled prevalence of 25 vs 40% for brain fog, 20 vs 40% for anxiety, a difference that was not observed with cognitive dysfunction. This suggests a subset of subjective cognitive complaints and psychiatric symptoms may in fact emerge *de novo* after the convalescent phase, and are likely not a direct consequence of acute COVID-19^82^. Objective cognitive dysfunction is associated with delirium/encephalopathy at the acute stage of disease, and with markers of disease severity such as ICU admission, even after propensity score matching^65^. Patients with cognitive dysfunction post-COVID should be evaluated for other causes of cognitive decline and dementia and treated accordingly, as impaired cognition is more common in older patients with pre-existing symptoms. There is currently no specific treatment for Long-COVID cognitive impairment, but patients may benefit from cognitive training^83^. 

Psychiatric syndromes showed a much less robust association with acute disease severity. A study by the HCFMUSP COVID-19 Study Group showed a composite of ‘mental and cognitive impairment’ was very common in hospitalized patients after six months, but was not related to baseline disease severity^81^. The partial dissociation between acute disease and neuropsychiatric symptoms suggests contextual psychological stressors play a major role. These include social isolation, confinement, post-traumatic stress, and regional characteristics, including a response to the striking neglect of the pandemic promoted by the Brazilian government^84^. This contrasts with fierce claims of universal organicity from journalists^85^ and patients online, as clearly demonstrated in an enlightening episode: in January 2021, Paul Garner, a professor of infectious diseases at the Liverpool School of Tropical Medicine, reported his own experience in overcoming Long COVID. He emphasized the similarity between Long COVID and Myalgic Encephalomyelitis/Chronic Fatigue Syndrome (ME/CFS), the role of self-help groups, refraining from seeking disease information on the internet, and “by retraining the bodily reactions with my conscious thoughts, feelings, and behaviour”^86^. The blog post and an accompanying tweet were met with intense discussion and overwhelmingly negative reactions from Long-COVID patients. One user wrote: “It’s a great shame that Prof Garner, who was a beacon in the early days of covid last Spring, when no-one of any note was validating the experience of ‘long covid’ sufferers, has chosen now to suggest it can all be cured by positive thinking”^86^. Several comments criticized Gardner’s comparison of Long-COVID and ME/CFS, frowning upon a possible suggestion of Long-COVID sharing the status of non-organic disease that many attribute to ME/CFS^55^. This explains why some skeptical doctors fear vilification as “medical gaslighters”^87^.

In conclusion, the COVID-19 pandemic brought major challenges to clinicians and neurologists. It illustrates how the production of medical knowledge is affected: by resource limitation; by the fear and excitement towards novelty (as shown by the surge of “neuro-COVID” case reports); and by the mobilization of subjective evidence and co-production of knowledge by patients and doctors. Social media and patient-led research introduced much welcomed and fast-paced patient-generated data, the downsides being an increase in selection bias and political pressure against clinicians and scientists. In the aftermath of the pandemic, we must be ready to provide care for a large mass of Long-COVID patients in all their physical, cognitive and psychological complexity.
